# Evolutions in the management of non-small cell lung cancer: A bibliometric study from the 100 most impactful articles in the field

**DOI:** 10.3389/fonc.2022.939838

**Published:** 2022-08-17

**Authors:** Siyuan Chen, Yu Qiao, Juan Chen, Yanan Li, Jianlian Xie, Pengfei Cui, Ziwei Huang, Di Huang, Yiming Gao, Yi Hu, Zhefeng Liu

**Affiliations:** ^1^ Department of Medical Oncology, Senior Department of Oncology, The Fifth Medical Center of PLA General Hospital, Beijing, China; ^2^ Medical School of Chinese PLA, Beijing, China; ^3^ Department of Medical Oncology, The First Affiliated Hospital of Bengbu Medical College, Bengbu, China; ^4^ School of Nursing, Yangzhou University, Yangzhou, China; ^5^ Department of Clinical Oncology, The Chinese University of Hong Kong, Hong Kong, Hong Kong SAR, China

**Keywords:** non-small cell lung cancer (NSCLC), management, treatment, bibliometric, R, VOSviewer, Citespace

## Abstract

**Objective:**

The study was designed to explore the evolution of non-small cell lung cancer (NSCLC) management in the last 20 years.

**Methods:**

The top 100 most-cited papers on NSCLC treatment were retrieved from the Web of Science Core Collection database. R and VOSviewer were used to extract bibliographic information, including the year of publication, countries/regions, institutions, authors, journals, keywords, impact factor, and total citations. The topic and type of papers were checked independently by authors. Bibliometric analysis was conducted and visualized with R, CiteSpace, Excel and VOSviewer to identify output dynamics, research forces, topics, hotspots, and frontiers in the field.

**Results:**

The average citation of each retrieved top 100 most-cited NSCLC management papers was 1,725 (range: 615-7,340). Fifty-seven corresponding authors were from the United States. This country contributed the most papers (n=76), followed by Germany (n=34), France (n=33), and South Korea (n=32). The top contributors were Paz-Ares L. (n=12) and Reck M. (n=12). The Memorial Sloan Kettering Cancer Center published the largest number of papers (n=20). There were two significant citation paths, indicating publications in medicine/medical/clinical journals primarily cited journals in molecular/biology/genetics fields, partly cited health/nursing/medicine fields. Top-cited papers mainly came from the New England Journal of Medicine (n=33, citations=80,427), followed closely by the Journal of Clinical Oncology (n=28, citations=32,408). “Chemotherapy” (n=36) was the keyword with the greatest frequency of co-occurrence. “Open-label” was the keyword with the strongest burst strength (=4.01), followed by “nivolumab” (=3.85), “blockade” (=2.86), and “efficacy” (=2.85).

**Conclusions:**

The United States as a nation and the Memorial Sloan Kettering Cancer Center as an institute contributed the most to this field. The New England Journal of Medicine is the most eye-catching journal. Hotspots of NSCLC management have almost undergone an evolution from chemotherapy and radiotherapy to targeted therapy to immunotherapy. Molecular/biological/genetic fields become the main research base for NSCLC treatment. Immunotherapy and combination therapy are research frontiers.

## Introduction

Lung cancer is the second most prevalent cancer and the most common cause of cancer-related deaths, with approximately 2.2 million new cases and 1.8 million deaths each year, accounting for one-tenth of diagnosed cancers and one-fifth of cancer deaths globally. This cancer has become the leading burden on worldwide health care (1). Non-small cell lung cancer (NSCLC) is the most common type of lung cancer, accounting for approximately 85% of all lung cancer cases, which is further subdivided into three types: adenocarcinoma, squamous carcinoma, and large cell lung carcinoma (2, 3). The most common type is adenocarcinoma, which occurs in the peripheral bronchi and accounts for approximately 40% of all lung cancers. This is followed by squamous carcinoma, which arises in the main bronchi and comprises 25-30% of all diagnosed lung cancers. Large cell carcinoma accounts for 10%, occurring in the proximal part within the thorax (3, 4). NSCLC is a heterogeneous malignancy harboring a wide variety of driver genetic mutations. Over the past few decades, tremendous advances in NSCLC treatment, especially the advent of targeted and immunotherapy, have changed the landscape of NSCLC management. Individualized precision medicine based on genetic characteristics is gaining popularity. Currently, the options for NSCLC management mainly include surgery, radiotherapy, chemotherapy, targeted therapy, and immunotherapy. Among them, surgery has become a greatly recommended choice for resectable NSCLC, stereotactic ablative radiotherapy (SABR) techniques make radiotherapy more precise and less damaging, platinum-based chemotherapy remains the standard regimen for some advanced NSCLC patients, while small molecular tyrosine kinase inhibitors (TKIs) and immune checkpoint inhibitors (ICIs) have brought unprecedented benefit in particular patients (5–7). The emergence of new technologies and the obsolescence of old ones have been ongoing.

However, the evolution of NSCLC treatment over the last 20 years is not well defined previously. Textbook-style chapter summaries and systematic reviews fail to fully demonstrate a time-based research progress and to effectively analyze a large amount of data. In fact, it is difficult for scholars to perform an exhaustive analysis in the face of the vast amount of NSCLC treatment research findings over the last 20 years. It is even more challenging to assess the evolution of the subject according to its temporal dynamics. Bibliometric analysis is one of the best tools for studying temporal trends in a certain area, which offers more comprehensive and objective results (8–11). Therefore, bibliometrics may provide meaningful insight into NSCLC management. We have innovatively applied this approach in the field of NSCLC treatment. The volume of citations in a paper signifies the significance of the study, indicating the impact it has had on the understanding and treatment of the disease (12). To make the study representative, we filtered the 100 most-cited articles in NSCLC treatment based on bibliometric citation analysis. These articles stand for the most impressive achievements in NSCLC treatment. In the 21st century, when the treatment of NSCLC is rapidly evolving, all research is inseparable from the foundation of previous generations. We can gain a lot of inspiration and experience from the evolution of NSCLC treatment. The study may help clinicians and researchers quickly understand the evolution of NSCLC management and grasp research status quo. The data visualization can help them to have a more intuitive understanding of the 100 most-cited NSCLC papers. To our knowledge, this is the first comprehensive bibliometric study about the evolution of NSCLC treatment in the last 2 decades.

## Materials and methods

### Data acquisition

The relevant data used in this study were downloaded from the Science Citation Index Expanded (SCI-EXPANDED) of Clarivate Analytics Web of Science Core Collection (WoSCC). The WoSCC is the most commonly used database for various bibliometric studies (8, 13). As the most prestigious global database, it can provide detailed information needed for bibliometric software and ensure the quality of research (14).

**Figure 1:**
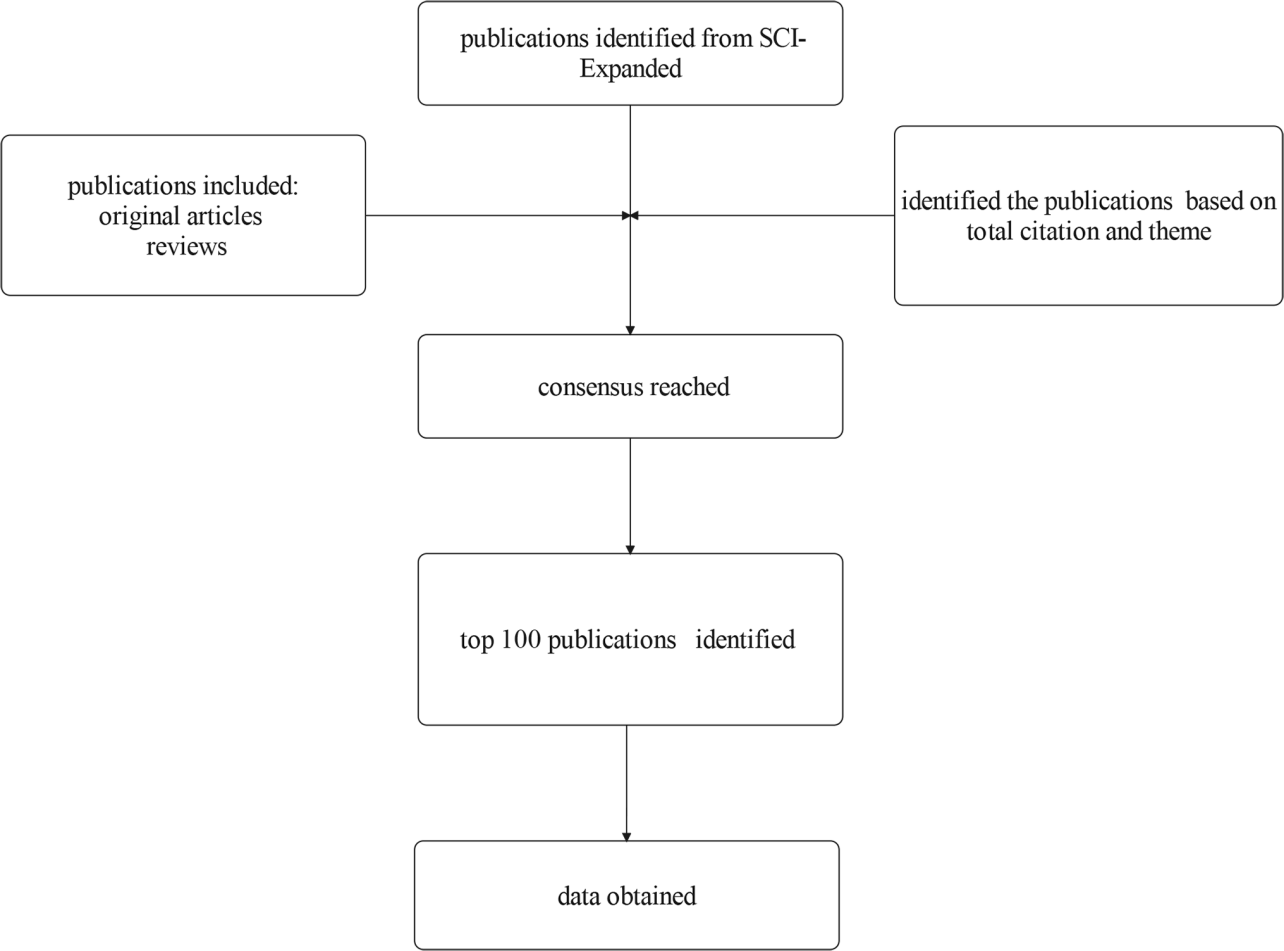
Flow chart of literature screening.

Literature in WoSCC published from January 2000 to December 2021 was systematically searched. The search terms were partly selected from the Medical Subject Headings (MeSHs) offered by the PubMed. We mainly refer to papers to expand the search terms (8). In addition, we consulted experts and physicians to add supplementary concept. The search topic terms were “non-small cell lung cancer” and “treatment”. The summary of the search strategy is presented in **Supplementary Table S1**. Guidelines, editorials, and statements were all excluded. Only original articles and reviews with full manuscripts regarding the management of NSCLC were reserved, and no language restrictions were applied. It has been demonstrated that bibliometric analysis using original articles and reviews is effective (15). To select the papers with the highest academic impact in the field, two authors (S.C. and Y.Q.) identified the top 100 articles based on total citation (TC) independently. If there was any dissensus, it was discussed with a corresponding expert (Y.H.) until a consensus was reached. To avoid errors caused by the database update, all data acquisition was completed on December 2, 2021. We download the 100 most influential articles’ records, in the “Full Record and Cited References” form from WoSCC in.txt format (**Figure 1**).

### Data analyses and visualization

R is a unique language and environment for statistical calculation and plotting (16). It offers a wide diversity of statistical and charting techniques and is highly extensible and useful in ever-changing fields, such as bibliometrics (11, 17). The “bibliometrix” R-package provides a range of flexible tools for quantitative research in bibliometrics and scientometrics while it can also be integrated with other R packages (11). We mainly rely on the “bibliometrix” package in the R software to convert, analyze, and visualize data.

VOSviewer, a computer program developed by Leiden University, used to build and view bibliometric maps, has exceptional capabilities in exploring and visualizing network-based data (18). It was chosen because it delivers concise, information-rich charts that meet the research needs.

CiteSpace, a JavaScript-based application developed by Drexel University, was designed as a powerful tool for identifying and mapping potential trends and dynamics of a scientific field over time (19). The program has been commonly applied for bibliometric analysis in numerous subjects, such as oncology, immunology, and regenerative medicine (9, 13, 20, 21).

The results of the bibliometric analysis are always manifested through science mapping, which is complex and frequently requires a combination of various software for the creation (11). The above applications were utilized in combination to achieve enriched results. We used the “bibliometrix” package (Version 3.1.4) in R software (Version 4.1.1) and VOSviewer (Version 1.6.17) to extract the bibliographic information of the selected papers, including the year of publication, countries/regions, institutions, authors, journals, keywords, impact factor (IF, from Web of Science InCites Journal Citation Reports, JCR Year 2020), and TC. To ensure the accuracy of the data, we also finalized the papers’ topic and type by reading titles, abstracts, and even the full texts. After that, we combine R and Microsoft Office Excel (Version 2019) to create the map concerning the annual distribution of publications, the proportion of international cooperation, the annual output of the top 10 authors, and the annual growth trends of the journals. VOSviewer was also used for graph generation of the overlay visualization map of the top-cited publications, the cooperation relationships of countries/regions, the item density visualization map of major institutions, the cooperation relationships of major authors, and the overlay visualization map of keywords co-occurrence. CiteSpace (Version 5.8.R3) was adopted to draw the dual-map overlay of journals and to identify the keywords with the strongest burst strength. Excel was used to manage the database.

## Results

### Output of publications

More than 300,000 articles (published between January 2000 and December 2021) related to the treatment of NSCLC were retrieved from WoSCC. The result shows that the TC varied from 615 to 7,340 and the average citations per publication were 1,725. These papers, including 97 original articles and 3 reviews, were published by a sum of 1,940 authors, with an average of 19.7 authors per publication, and none of the papers were solely authored.

**Figure 2:**
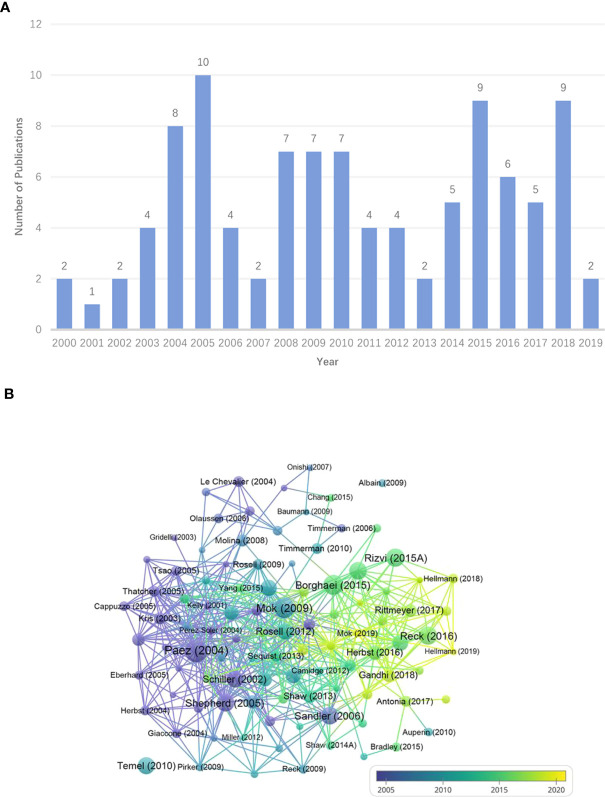
**(A)** Annual distribution of publications. **(B)** The overlay visualization map of the top-cited publications. Each node means an article. The size of circles and fonts is proportional to TC. The more purple the node, the earlier the year of publication, and the yellower the node, the more recent the publication date. The node name shows the first author and the publication year of a paper. The connecting line suggests that there is a citation relationship between the papers.

As is displayed in **Figure 2A**, the selected most influential papers were published from 2000 to 2019, with annual output ranging from 1 to 10. The high-yield years were 2005 (n=10), 2015 (n=9) and 2018 (n=9) and the lowest yield year was 2001 (n=1).

**Figure 2B** reflects that the single papers published by Paez JG, Mok TS, and Borghaei H had the top 3 TC, with 7,340, 5,931, and 5,453 respectively. In addition, there was a wide citation relationship between the top papers. The detailed information about the 100 most influential publications was recorded in **Supplementary Table S2**.

### Countries/regions analysis

**Figure 3:**
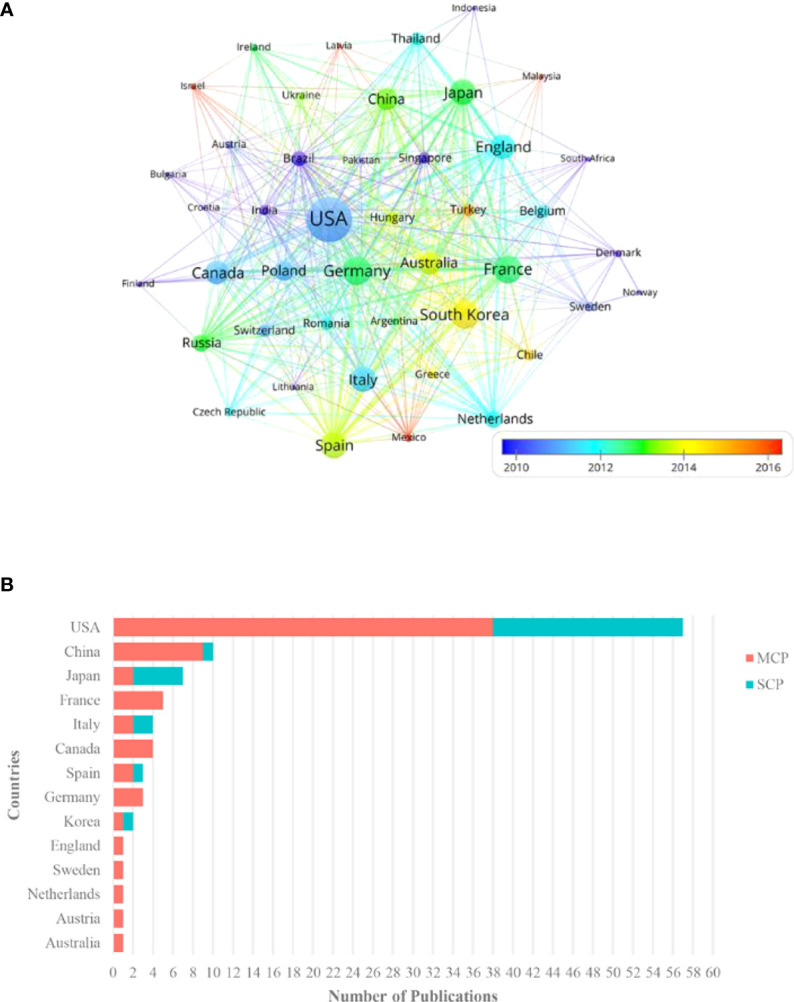
**(A)** The cooperation relationships of countries/regions. Each node represents a country/region. The size of circles and fonts symbolizes the number of articles in certain countries/regions, and the thickness of the linking line between countries/regions indicates the frequency of collaborations. The distance between the two circles demonstrates the relatedness of their link. **(B)** The proportion of international cooperation. The length of the bar is determined by the number of corresponding authors in the country/region. MCP is intercountry collaboration indices, denoting the number of papers issued collaboratively by multiple countries/regions. SCP is intra-country collaboration indices, indicating the number of papers published independently by a single country/region.

As is shown in **Figure 3A**, the 100 top-cited articles were from 44 countries/regions. Among these countries/regions, the United States possesses the largest weight (n=76), meaning the number of papers involving authors from the United States was 76. The densest connecting lines surrounding “Unites States” suggests the strongest cooperation between the United States and other countries/regions. The highly cooperative countries/regions also include Germany (n=34), France (n=33), South Korea (n=32), Spain (n=29), Italy (n=27), Japan (n=27), United Kingdom (n=25), Canada (n=24), Australia (n=22) and China (n=21) (shown in the **Supplementary Table S3**).

**Figure 3B** demonstrates the distribution of corresponding authors. The level of scientific research in a country/region depends to a large extent on the number of experts in the field. Generally, the corresponding author is the leader in charge of the research and the gatekeeper of the final quality of academic papers. To further explore leading countries/regions in the area, we analyze the distribution of corresponding authors. The majority of the article’s corresponding authors are from the United States (n=57), followed by China (n=10), Japan (n=7), France(n=5), Italy (n=4), Canada (n=4), Spain (n=3), Germany (n=3) and Korea (n=2).

### Institutions analysis

**Figure 4:**
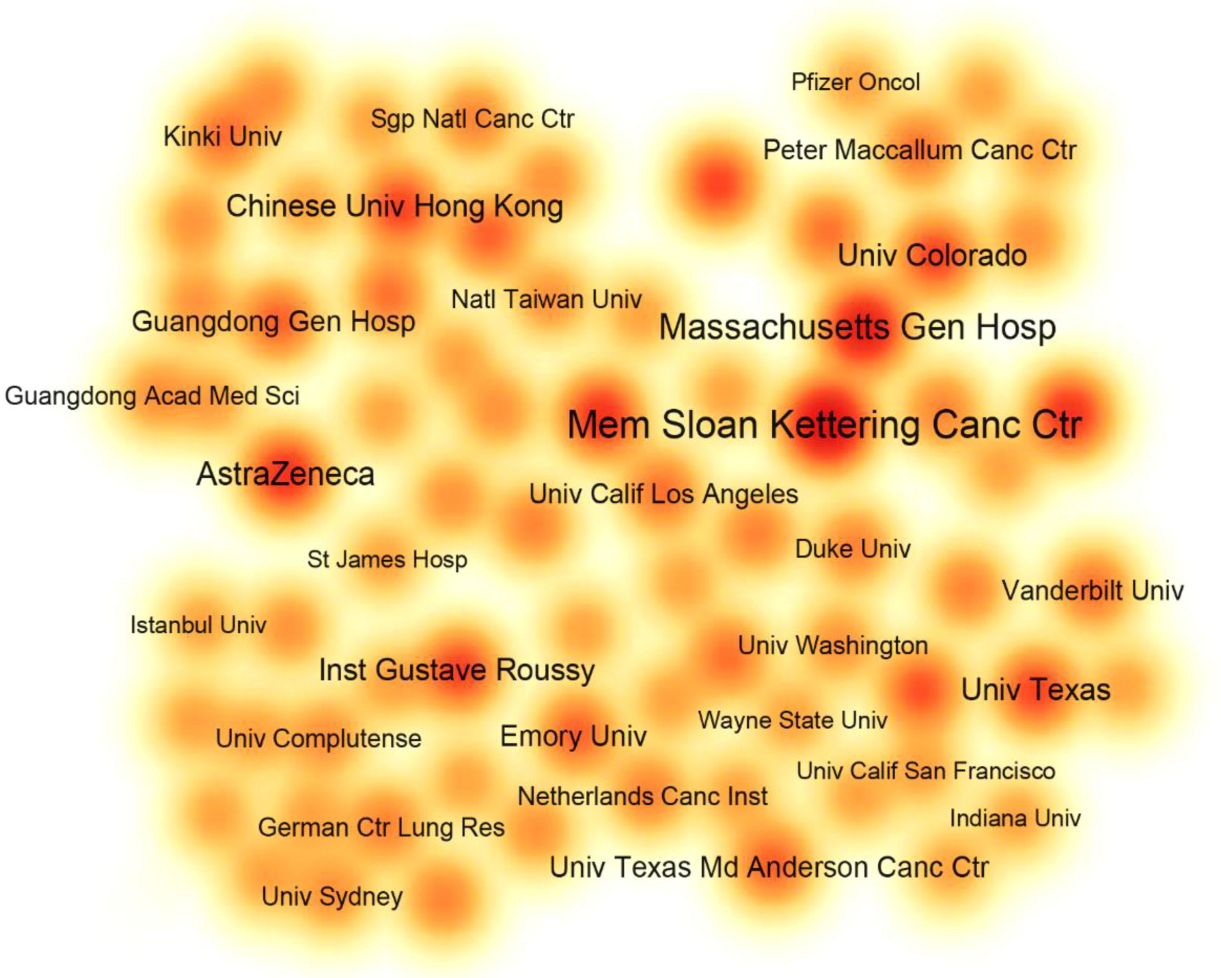
The item density visualization map of major institutions. Each node represents an institution. The larger the size of the fonts and the closer the color around the node to red, the more papers the institution is involved in.

As is noted in **Figure 4**, The Memorial Sloan Kettering Cancer Center holds the largest weight (n = 20), which signifies that the institution was involved in publishing 20 papers, followed by the Massachusetts General Hospital, the AstraZeneca, the Dana-Farber Cancer Institute, and the Sungkyunkwan University, with 16, 12, 12 and 12 respectively. TC/publication is the ratio of TC to number of publications and is equal to the average TC per paper, reflecting the average impact of papers in the journal.

As is listed in **Table 1**, the Dana-Farber Cancer Institute had the highest TC/publication (2,635.00), followed by the AstraZeneca (1,909.00), the Chinese University of Hong Kong (1,760.20), and the Memorial Sloan Kettering Cancer Center (1,747.15).

**Table 1:** Top 10 institutions with the most articles.

Rank	Institutions	Country/Region	Publication	TC	TC/Publication
1	The Memorial Sloan Kettering Cancer Center	USA	20	34943	1747.15
2	The Massachusetts General Hospital	USA	16	27151	1696.94
3	The AstraZeneca	England	12	22908	1909.00
4	The Dana-Farber Cancer Institute	USA	12	31620	2635.00
5	The Sungkyunkwan University	South Korea	12	19163	1596.92
6	The Chinese University of Hong Kong	China	10	17602	1760.20
7	The Institute Gustave Roussy	France	10	16688	1668.80
8	The Seoul National University Hospital	South Korea	10	13958	1395.80
9	The University of Colorado	USA	10	15836	1583.60
10	The University of Texas	USA	10	12761	1276.10

### Authors analysis

**Figure 5:**
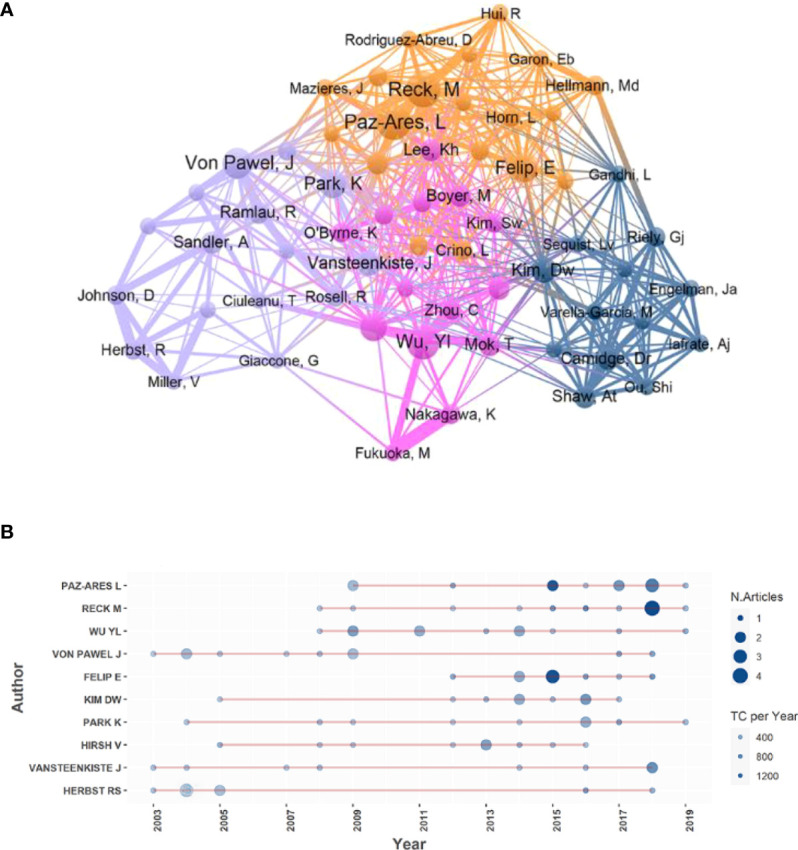
**(A)** The cooperation relationships of major authors. Each node stands for an author. The size of circles and fonts means the articles’ counts of the author. The colors of the nodes represent clusters. The thickness of the connecting line between authors shows the frequency of collaborations. The distance between the two circles expressed the relatedness of their link. **(B)** Annual output distribution of the top 10 authors. The bigger the node, the more papers published in that year, and the bluer the node, the more annual average TC of the authors’ papers in that year.

In **Figure 5A**, major authors were divided into 4 clusters, which can help us identify the core research teams. The researchers formed a collaborative network with Paz-Ares L., Reck M., Wu Yl., Von Pawel J., and Felip E. as the core, each publishing 12, 12, 11, 10, and 9 articles.

As is shown in **Figure 5B**, there was a large gap (nearly 10 years) between the two top articles published by Herbst R. whereas some authors, like Paz-Ares L. and Reck M., had been publishing articles for years in a row.

### Distribution of journals

**Figure 6:**
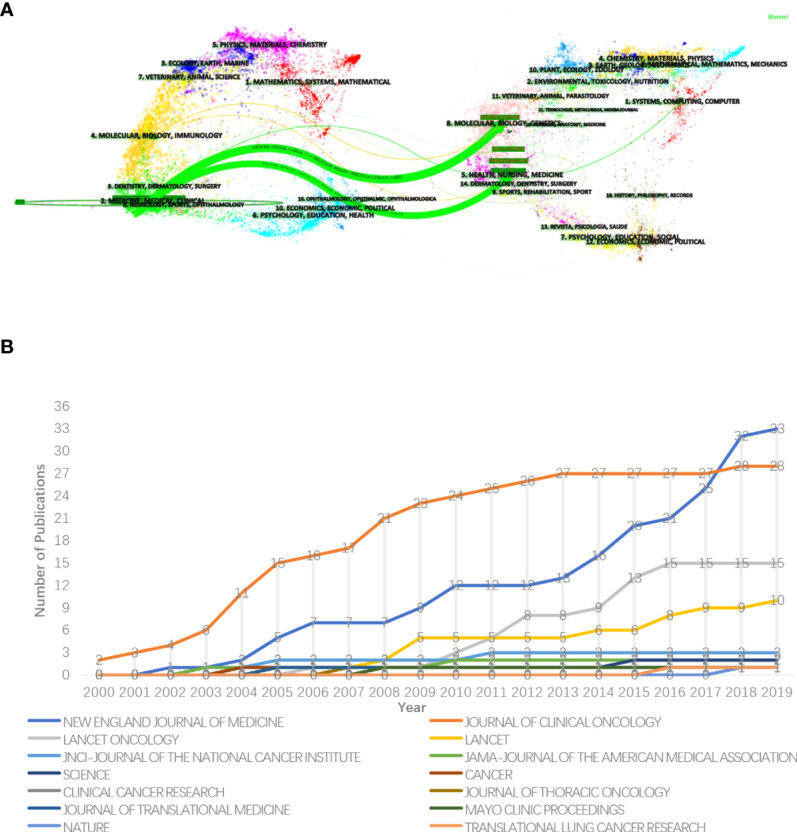
**(A)** The dual-map overlay of journals. The citing papers are listed on the left while the cited papers are laid on the right, between them was the curve that presents the citation relationship. Different colors denote journals from different subjects. The length of the vertical axis of the ellipse is proportional to the papers’ counts published in the journal and the horizontal length is to authors. **(B)** The annual growth trends of the journals.

**Figure 6A** conveys the category distribution of the journals (22). Two significant citation paths were identified and marked in green. The first green path indicates publications in medicine/medical/clinical journals primarily cited journals in molecular/biology/genetics fields and the second means publications in medicine/medical/clinical journals partly cited health/nursing/medicine journals. Immunology and computer-related journals also appear in the citation path marked in yellow and green.

From **Figure 6B**, the 100 most impactful papers were published in 14 journals. Source dynamics show that most of relevant papers (n=33) were published in the New England Journal of Medicine and had an exponential-like growth in recent years (2012–2019), followed by the Journal of Clinical Oncology (n=28), the Lancet Oncology (n=15) and the Lancet (n=10).

The details are listed in **Table 2**. The New England Journal of Medicine had the highest IF (=91.253) and TC (=80,427). The 3 journals with the ratio of TC to publication over 2,000 were the Science (=5,933.00), the New England Journal of Medicine (=2,437.18), and the Mayo Clinic Proceedings (=2,049.00).

**Table 2:** Journals that published the most-cited 100 publications.

Journal	Publication	IF	TC	TC/Publication
New England Journal of Medicine	33	91.253	80427	2437.18
Journal of Clinical Oncology	28	44.544	32408	1157.43
Lancet Oncology	15	41.316	20771	1384.73
Lancet	10	79.323	14156	1415.60
JNCI-Journal of the National Cancer Institute	3	13.506	2672	890.67
JAMA-Journal of the American Medical Association	2	56.274	3824	1912.00
Science	2	47.728	11866	5933.00
Cancer	1	6.86	623	623.00
Clinical Cancer Research	1	12.531	685	685.00
Journal of Thoracic Oncology	1	15.609	688	688.00
Journal of Translational Medicine	1	5.531	628	628.00
Mayo Clinic Proceedings	1	7.619	2049	2049.00
Nature	1	49.962	1112	1112.00
Translational Lung Cancer Research	1	6.498	623	623.00

### Keywords, topics and frontiers

The theme of a paper is reflected in the keywords, and by analyzing them, researchers can get an idea of the topic of the article (23). The presence of two keywords from a certain field in the same article, called co-occurrence, reflected that there is some internal relationship between them, and the more they appear, the closer the relatedness is (24).

**Figure 7:**
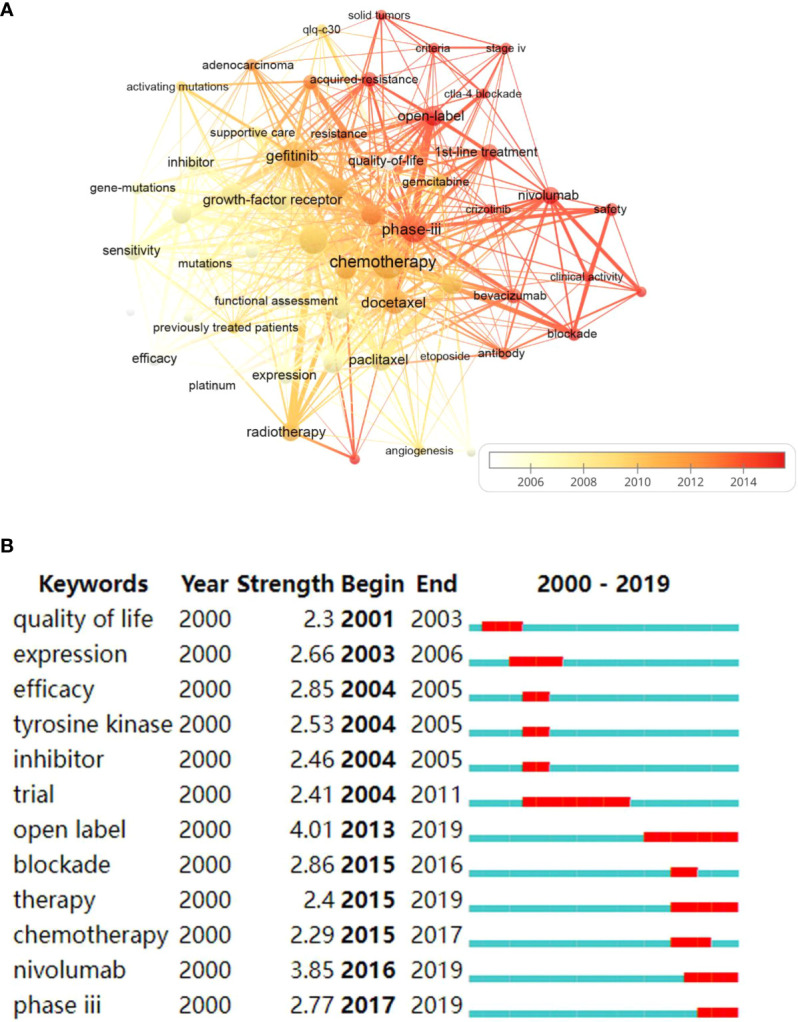
**(A)** The overlay visualization map of keywords co-occurrence. Each node represents a keyword. The size of circles and fonts is proportional to the frequency of keywords. The thickness of connecting lines stands for the co-occurrence frequency. The whiter the node is, the earlier the focus on this topic, and the redder is, the more attention it gets nowadays. **(B)** Keywords with strongest burst strength. The red bars indicate the sudden increase of occurrence frequency of the keyword in this period and the blue ones denote the unpopular period.

The keyword co-occurrence network (**Figure 7A**), based on the above principles, can detect the research dynamic and structure of the discipline (25). Keyword plus terms of WoS are effective and more broadly descriptive (26). We chose it to perform keyword analysis. The densest connecting lines around the keyword “chemotherapy” indicate that it is most closely related to other keywords.

As is listed in **Table 3**, the keywords with the highest frequency were “chemotherapy” (n=36), followed by “clinical-trials” (n=28), “phase-iii” (n=24), and “gefitinib” (n=22).

**Table 3:** Top 20 keywords with the most occurrence.

Rank	Keywords	Counts	Rank	Keywords	Counts
1	Chemotherapy	36	11	Erlotinib	14
2	Clinical-trials	28	12	Combination	13
3	Phase-iii	24	13	Survival	13
4	Gefitinib	22	14	Tyrosine kinase inhibitor	13
5	Docetaxel	19	15	Open-label	12
6	Growth-factor receptor	15	16	Multicenter	11
7	Paclitaxel	15	17	Radiotherapy	11
8	Carboplatin	14	18	Sensitivity	10
9	Cisplatin	14	19	Nivolumab	9
10	EGFR	14	20	1st-line treatment	8

**Figure 7B** shows the keywords with the strongest burst strength. Burst-detection algorithms can recognize emerging terms regardless of the number of citations in the host articles, so that burst terms can sensitively and accurately capture the research frontiers (19). Keywords burst earlier indicate that researchers focused on this area in early years, while burst closer to the present denote the topic has suddenly attracted attention recently. The top 5 keywords identified are “open-label”, “nivolumab”, “blockade”, “efficacy” and “phase iii”, with the burst strength of 4.01, 3.85, 2.86, 2.85, and 2.77, respectively. “Quality of life” is the earliest keyword to burst, and the most recent burst keywords include “phase iii” and “nivolumab”. Keywords with the longest duration of burst are “trial” and “open label”.

### Clinical application

**Figure 8:**
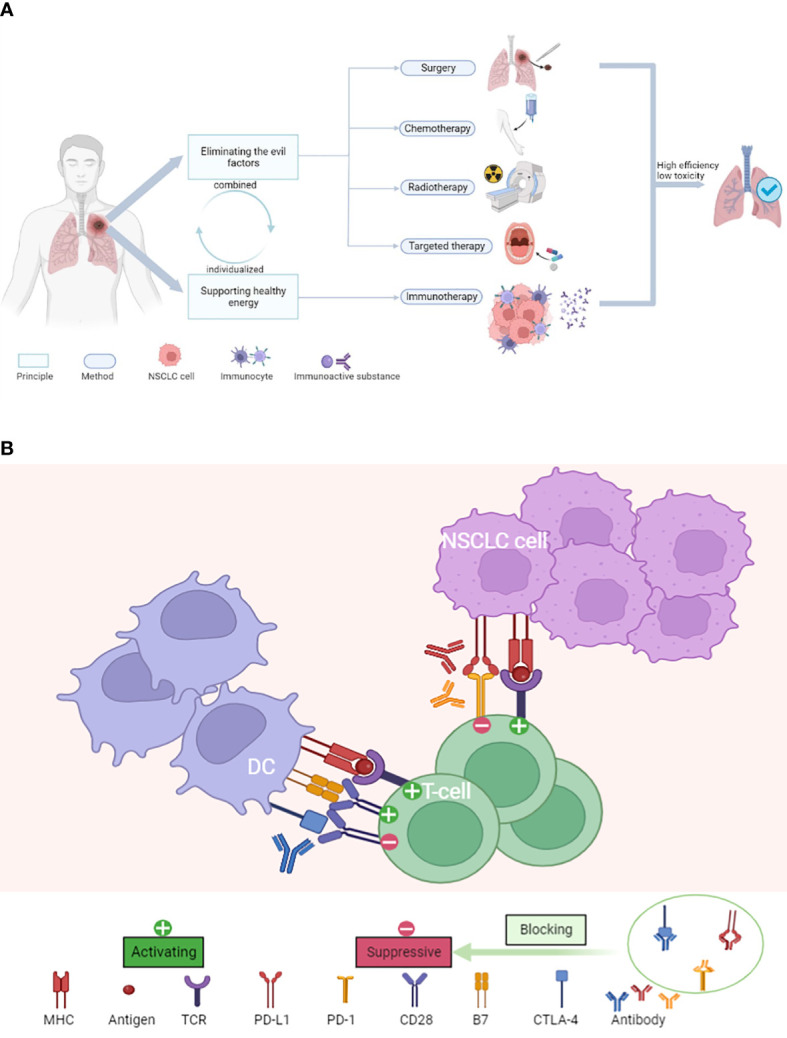
**(A)** The principles and landscape of NSCLC management. **(B)** The major mechanism of ICIs for the treatment of NSCLC.

**Figure 8A** exhibits the principles and landscape of NSCLC management. Chemotherapy, radiotherapy, surgery, targeted therapy and immunotherapy are the main regimens of current NSCLC treatment and have been widely used in clinical practice.

As shown in **Figure 8B**, ICIs, as new therapeutic agents, have unique advantages, and the mechanism of the ICIs is more in line with the future research direction.

## Discussion

With the significant advancements and rapid changes in NSCLC treatments in the last 20 years, it is crucial to understand the progress and evolution of NSCLC management in the new era where many therapies coexist (5). However, available textbooks and papers lack such information. We selected 100 most impactful papers in this field as research data and applied the bibliometric analysis to systematically explore the output dynamics, research forces, topics, hotspots, and frontiers in the field of NSCLC treatment. It can help scholars quickly learn the basics, clarify study ideas, and learn the research status quo.

As can be seen from **Figure 3**, top articles evidenced the merits of international cooperation and witnessed a multi-national cooperation network with the United States as the core. Half of the top 10 institutions are from the United States (rank 1, 2, 4, 9, 10 in **Table 1**). There are 57 corresponding authors belonging to the United States, reflecting the United States is the originator of most top papers and has the most specialists. Additionally, some European countries such as Germany, Britain, and Italy, Canada in North America, and China, Japan, and Korea in Asia, also have outstanding performance in the field In 2000, a phase III clinical trial conducted in the United States demonstrated for the first time that docetaxel monotherapy versus vinorelbine/ifosfamide could provide clinical benefit for patients with advanced NSCLC who had relapsed or progressed after receiving platinum-based chemotherapy (27). A phase II study performed in the United States in 2004 investigated the clinical efficacy of erlotinib and found that rash might be a clinical marker for efficacy prediction (28). About a decade ago, this country explored the toxicity and efficacy of stereotactic body radiation therapy in patients with early-stage inoperable NSCLC and concluded that the therapy had high local control rates and moderate adverse events (29). In recent years, the country has also made significant achievements in immunotherapy, such as a single-arm clinical trial covering 27 sites in the United States, France, Germany, and Italy, which revealed favorable efficacy and safety of nivolumab in previously treated patients with refractory advanced squamous NSCLC (30). The United States leads all areas of NSCLC treatment in the world.

As illustrated in **Figure 4**, the Memorial Sloan Kettering Cancer Center is the foremost research institution for NSCLC management, with the most top-notch papers and highest TC, followed closely by Massachusetts General Hospital. AstraZeneca, the Dana-Farber Cancer Institute, and the Sungkyunkwan University also with exceptional academic results. These institutions all have a long history of distinguished contributions to the field of NSCLC therapy. We found that the major studies in which the Memorial Sloan Kettering Cancer Center was involved were divided into two parts: before 2015 the theme of research was targeted therapy, and after 2015 the topic was immunotherapy. In 2003, the institution participated in a phase II clinical trial that further demonstrated the benefit of gefitinib in improving post-chemotherapy NSCLC symptoms and inducing radiographic cancer regressions (31). In 2015, it was involved in a randomized, open-label, international phase III study comparing the efficacy of nivolumab and docetaxel in patients with non-squamous NSCLC that progressed during or after platinum-based dual chemotherapy, proved that nivolumab had longer overall survival (32). The study has been cited more than 5,000 times.

The major contributors (shown in **Figure 5**), such as Paz-Ares L., Reck M., and Wu Yl., come from different countries and are affiliated with different institutions. It is their long-lasting efforts and closer global collaboration that have driven the development of NSCLC treatment. We found that the distribution of the authors’ top papers seemed no correlation with time. Interestingly, both Paz-Ares L. and Reck M. had published remarkable results in 2018, and the topics were both centered on immunotherapy for NSCLC (33, 34). It was also in 2018 that the Nobel Prize in Physiology or Medicine Prize was granted to scientists James P. Allison (United States) and Tasuku Honjo (Japan) for their pioneering work in tumor immunotherapy. Thus, we assume that 2018 was an important time-point for NSCLC immunotherapy.

The distribution of the journals (**Figure 6A**) demonstrates the treatment of NSCLC is increasingly reliant on molecular, biological, and genetic fields. Thanks to breakthroughs in targeted therapeutic pathways, different targeted regimens have been applied to patients with different genetic mutations, such as EGFR-positive and ALK-positive mutations. Furthermore, that advancement can predict efficacy, safety, and prognosis of targeted therapy (35–37). The molecular mechanism of immune checkpoint blockade has been partially elucidated. Tumor mutation burden and PD-l expression levels were utilized to predict the outcome of immunotherapy (38, 39). The citations in journals related to immunology and computer science indicate that the field has moved toward multidisciplinary integration. Increasingly routine clinical application of targeted next-generation sequencing technology, immunohistochemical techniques, and comprehensive genomic profiling enables more precise NSCLC management and makes personalized management possible (38, 40, 41). Clinical trials and basic experiments are becoming growingly inseparable today, inaugurating the century of rapid advancement in NSCLC management.

From **Figure 6B**, the most authoritative journal in the field is the New England Journal of Medicine, which has the most top-cited papers, the fastest papers growth rate, the highest IF and TC, followed by Journal of Clinical Oncology, Lancet Oncology, etc. Papers published in these journals are likely to be of higher academic quality. The most cited paper in the New England Journal of Medicine is about 6,000 times. The paper reported an open-label, phase III trial for non-smoking or formerly lightly smoking untreated patients with adenocarcinoma in East Asia. It demonstrated superior progression-free survival with gefitinib as initial treatment over carboplatin-paclitaxel in this population (42).

As is presented in **Figure 7A**, chemotherapy and targeted therapy have been studied in a lot of top papers, while the counts of immunotherapy-related keywords, such as nivolumab, were relatively small, probably because it as an emerging regimen has not been heavily cited yet. Of note, the earliest article was published in 2000 on the topic of chemotherapy in NSCLC (27), and the most recent 3 publications published in 2018 and 2019, all focused on immunotherapy (33, 43, 44). The timeline of the keywords illustrated that the hotspots of NSCLC management have evolved from chemotherapy and radiotherapy to targeted therapy and then to immunotherapy.

Surgery and cytotoxic drugs were introduced to treat NSCLC in the 1960 and 1970, making the first leap forward in NSCLC treatment. Surgery is currently the most recommended method for NSCLC patients with stage I-II, yet 70% of patients are in advanced stage III-IV at the time of diagnosis. Cytotoxic drugs, targeted therapy, and immunotherapy are critical for patients with advanced NSCLC (41). Among 100 highly cited papers, there is no research with the theme of surgery for NSCLC, which may be related to the high maturity and effectiveness of video-assisted thoracic surgery (VATS) (45, 46). SABR is considered as a standard care for inoperable peripheral type early-stage NSCLC, showing meaningful clinical benefit (47). Over the past 21 years, the common cytotoxic chemotherapy, such as paclitaxel and docetaxel, remains the predominant therapy for advanced NSCLC to some extent, but with the development of targeted therapy, TKIs have become the first-line treatment for some driver gene mutation-positive NSCLC patients.

TKIs such as gefitinib, and anti-angiogenic agents such as bevacizumab, were approved by the Food and Drug Administration (FDA) for NSCLC treatment. New targeted therapeutics are continually being approved by the FDA. In 2003, gefitinib was granted for treatment of second-line, unselected advanced NSCLC (48). Then in 2006, bevacizumab plus paclitaxel and carboplatin became the first-line regimen for non-squamous NSCLC, approved by the FDA. Using ALK-TKIs and EGFR-TKIs as first-line in the 2010 updates the options for the therapy of NSCLC (41). Third-generation targeted agents, such as osimertinib, and new combination regimens, for example EGFR-TKI erlotinib in combination with anti-angiogenic agent ramucirumab approved for EGFR-mutant NSCLC in 2020, were also gradually being used in the clinical practice (5, 41). Along with the application of targeted therapies, researchers were also more devoted to studying gene mutations and growth-factor receptors, which are inextricably linked to the effectiveness of targeted therapy (40). There is no doubt that targeted therapy is a milestone in the treatment of NSCLC.

In **Figure 7A**, the large number of lines between treatment modalities implied combination application to different degrees, and the density of the lines did not decrease over time, pointing that combination medication has always been a concern for researchers and remains a hot academic topic, based on which we recommend scholars make more attempts on combination therapy for NSCLC. Keywords with higher burst strength may become the new turning point (49, 50), which can lead us to find emerging hotspots and frontiers of the field. **Figure 7B** show that after the research boom of targeted therapy represented by TKIs, immune checkpoint blockade represented by programmed cell death-1 (PD1) and programmed cell death ligand-1 (PD-L1) blockade, such as “nivolumab” with the burst strength of 3.85, may become turning points in NSCLC treatment. Actually, cytotoxic T-lymphocyte antigen-4 (CTLA-4) and PD-1/PD-L1 blockade are research frontiers. In 2015, the FDA authorized nivolumab for the treatment of patients whose tumor had progressed on or after platinum-based regimens, a dramatic breakthrough in the treatment of advanced NSCLC that foreshadowed an immune era in lung cancer treatment (5, 51). In 2016, pembrolizumab was approved as monotherapy for first-line treatment of NSCLC with PD-L1 ≥ 50%. Pembrolizumab combined with chemotherapy as first-line treatment for advanced non-squamous NSCLC, granted in 2017, provides survival benefit for patients without target gene mutations (41). Furthermore, durvalumab became a new treatment option for adjuvant therapy in 2018. In 2020, first-line treatment using nivolumab plus ipilimumab and double-platinum chemotherapy for advanced NSCLC also proved the effectiveness of ICIs therapy (52). Another PD-1 inhibitor, such as cemiplimab-rwlc, was approved for first-line treatment in 2021, further confirming the rise of immunotherapy (41). Immunotherapy is perhaps the most significant breakthrough in NSCLC treatment in the last 20 years. It has profoundly changed our treatment landscape and research outlook. More immunologic agents will be available for NSCLC management, while the comparison between different immunologic agents may be worth the attention of researchers. Indeed, immunotherapy has shown excellent anti-tumor effects and a better prognosis than conventional therapy for patients with advanced NSCLC without EGFR or ALK mutations. Although patients can benefit from ICIs, most of patients may develop resistance after use, and combination therapy is considered a viable approach to overcome acquired resistance (53, 54). At present, ICIs are being explored as combination or monotherapy in neoadjuvant or adjuvant settings for NSCLC management and the results were promising (34, 39, 44). New immunotherapies, such as tumor vaccines, are being developed (3, 55). Researchers are also paying more attention to issues such as drug safety and resistance (30, 56). In terms of trial design, “open label” trials with the burst strength of 4.01, and “phase iii” clinical trials, 2.77, were widely used in the top articles. The large sample size, multicenter, international collaborative RCTs and experiments are necessary to derive reliable studies. Top researchers preferred to explore the efficacy of the combination of various regimens in different types of NSCLC while are more passionate about seeking effective first-line treatment alternatives. Recently, in top-cited papers, immunotherapy and chemotherapy were often combined to treat NSCLC patients or used separately to compare clinical value (33, 43), which is also consistent with **Figure 7B**, where “chemotherapy” and “nivolumab” red bars overlap in some years.

As shown in **Figure 8**, the treatment principles of diseases can be divided into two parts: eliminating the evil factors and supporting healthy energy, which is complementary to each other. We think that the management of lung cancer may also be divided based on this principle. Traditional therapy methods (surgery, chemotherapy, radiotherapy, targeted therapy) (54) kill tumor cells directly, in keeping with the principle of eliminating harmful factors. On the other hand, immunotherapy destroys tumor cells indirectly by triggering or improving the immune function of the body, supporting healthy energy. The search for more effective, safer, and sustainable first-line treatment options using a combination of chemotherapy, radiotherapy, surgery, targeted therapy, and immunotherapy is the focus of researchers and clinicians in nowadays. The potential of immunotherapy has not yet been fully explored, and a new therapeutic leap after it is yet to come. Nevertheless, researchers should actively consider what the next epoch-making breakthrough after immunotherapy will be and conceive the future landscape of NSCLC treatment. We speculate that this breakthrough should be based on genetic and molecular technology, relying on the body’s inherent anti-tumor ability rather than the direct killing of tumors, in line with the principle of supporting healthy energy.

### Limitation

This study has certain limitations. First, although we identified the most influential papers in NSCLC management based on bibliometric citation analysis and the broad search terms we have formulated, there is still a possibility of missing articles, such as recently published papers that may be widely cited at a future date but do not currently accumulate enough citations to be included in our data. To minimize information omission caused by publication time, we discussed the latest papers to derive the new research progress in NSCLC treatment. Second, our data are all from the WoSCC, so some publications indexed in other databases may be omitted. Databases such as Scopus or PubMed can be used in further research. Third, bibliometric tools inevitably exclude some secondary topics when mapping despite our combination of multiple tools to counteract this effect, and this information probably is also important. Nonetheless, unlike most papers using single tools, we innovatively combined R software, CiteSpace, VOSviewer, and Excel, which may help us achieve comprehensive insights from multiple viewpoints. To our knowledge, the study is the first comprehensive bibliometric assessment of the evolution of NSCLC treatment in the 21^st^ century.

## Conclusion

We found that the United States is the strongest research nation and the Memorial Sloan Kettering Cancer Center is the most prominent institution over the last 20 years. Core authors, such as Paz-Ares L. and Reck M., have developed extensive international collaborations. The New England Journal is the most authoritative journal in the field. Molecular, biological, and genetic research plays a progressively important role in the treatment of NSCLC, while immunology and computer science have also been widely applied to this field. Hotspots of NSCLC management have almost undergone an evolution from chemotherapy and radiotherapy to targeted therapy to immunotherapy, while the exploration of combination therapy has never stopped. Immunotherapy and combination therapy are research frontiers. Encouraging progress has been made in the management of NSCLC in the last 2 decades, and more breakthroughs are sure to be explored in the future.

Overall, our study revealed leading countries, core institutions, distinguished authors, authoritative journals, citation relationships, topic dynamics, and research frontiers in the field of NSCLC management. That information may help researchers quickly sort out the historical progress of NSCLC treatment, provide insight into the future advancement of the field, and guide future research practice.

## Data availability statement

The original contributions presented in the study are included in the article/**Supplementary Material**. Further inquiries can be directed to the corresponding authors.

## Author contributions

ZL and SC designed the study. SC wrote the manuscript. SC, YH, and YQ selected the 100 most impactful papers and did the bibliometric analysis. YQ and JC guided the use of the software. SC, YQ, and JC framed the article. YL, JX, and PC proposed constructive opinions. ZH, DH, and YG helped retrieve data. ZL and YH led the team and revised the paper. All authors conducted the literature search and analyzed the data. All authors contributed to the article and approved the submitted version.

## Conflict of interest

The authors declare that the research was conducted in the absence of any commercial or financial relationships that could be construed as a potential conflict of interest.

## Publisher’s note

All claims expressed in this article are solely those of the authors and do not necessarily represent those of their affiliated organizations, or those of the publisher, the editors and the reviewers. Any product that may be evaluated in this article, or claim that may be made by its manufacturer, is not guaranteed or endorsed by the publisher.
