# Supplementary material for: Evolutions in the management of non-small cell lung cancer: A bibliometric study from the 100 most impactful articles in the field
Source: Front Oncol. 2022 Aug 17;12:939838. doi: 10.3389/fonc.2022.939838 (PMC9428518; doi:10.3389/fonc.2022.939838)
Supplement: Supplementary file 1 [file DataSheet_1.zip › Additional files/Figure production/CiteSpace figure production/project/CO-DESC_history_view_burstness_ByStartingYear.html]

View Citation Burst History

# Top 12 Keywords with the Strongest Citation Bursts

Keywords | Year | Strength | Begin | End | 2000 - 2019 || quality of life | 2000 | 2.3 | **2001** | 2003 | ▂▃▃▃▂▂▂▂▂▂▂▂▂▂▂▂▂▂▂▂ |
| expression | 2000 | 2.66 | **2003** | 2006 | ▂▂▂▃▃▃▃▂▂▂▂▂▂▂▂▂▂▂▂▂ |
| efficacy | 2000 | 2.85 | **2004** | 2005 | ▂▂▂▂▃▃▂▂▂▂▂▂▂▂▂▂▂▂▂▂ |
| tyrosine kinase | 2000 | 2.53 | **2004** | 2005 | ▂▂▂▂▃▃▂▂▂▂▂▂▂▂▂▂▂▂▂▂ |
| inhibitor | 2000 | 2.46 | **2004** | 2005 | ▂▂▂▂▃▃▂▂▂▂▂▂▂▂▂▂▂▂▂▂ |
| trial | 2000 | 2.41 | **2004** | 2011 | ▂▂▂▂▃▃▃▃▃▃▃▃▂▂▂▂▂▂▂▂ |
| open label | 2000 | 4.01 | **2013** | 2019 | ▂▂▂▂▂▂▂▂▂▂▂▂▂▃▃▃▃▃▃▃ |
| blockade | 2000 | 2.86 | **2015** | 2016 | ▂▂▂▂▂▂▂▂▂▂▂▂▂▂▂▃▃▂▂▂ |
| therapy | 2000 | 2.4 | **2015** | 2019 | ▂▂▂▂▂▂▂▂▂▂▂▂▂▂▂▃▃▃▃▃ |
| chemotherapy | 2000 | 2.29 | **2015** | 2017 | ▂▂▂▂▂▂▂▂▂▂▂▂▂▂▂▃▃▃▂▂ |
| nivolumab | 2000 | 3.85 | **2016** | 2019 | ▂▂▂▂▂▂▂▂▂▂▂▂▂▂▂▂▃▃▃▃ |
| phase iii | 2000 | 2.77 | **2017** | 2019 | ▂▂▂▂▂▂▂▂▂▂▂▂▂▂▂▂▂▃▃▃ |
