# Supplementary material for: Evolutions in the management of non-small cell lung cancer: A bibliometric study from the 100 most impactful articles in the field
Source: Front Oncol. 2022 Aug 17;12:939838. doi: 10.3389/fonc.2022.939838 (PMC9428518; doi:10.3389/fonc.2022.939838)
Supplement: Supplementary file 1 [file DataSheet_1.zip › Additional files/Supplement Table S1-S3/Supplementary Table S1.docx]

**Table S1 | The summary of the keywords and search strategy to identify the top 100 articles from the Clarivate Analytics Web of Science Core Collection database.**

**#1:** (non-small cell lung cancer) OR (NSCLC) OR (non-small cell lung carcinoma) OR (non-small lung cancer) OR (non-small cell lung) OR (lung cancer) OR (non-small cell cancer) OR (none small cell lung cancer) OR (non-small cell) OR (squamous cell carcinoma) OR (adenocarcinoma) OR (large cell lung cancer)

**#2:** (Therapeutic) OR (Therapy) OR (Treat) OR (Treatment) OR (Management) OR (Surgery) OR (Surgical) OR (Operate) OR (operation) OR (resection) OR (lung resection) OR (lung Transplantation) OR (Transcatheter arterial chemoembolization) OR (Transarterial chemoembolization) OR (Transarterial embolism) OR (TAE) OR (Ablation) OR (Radiofrequency ablation) OR (Microwave ablation) OR (RFA) OR (Immunotherapy) OR (Chimeric Antigen Receptor T-Cell Immunotherapy) OR (Vaccine) OR (Targeted therapy) OR (Molecular Therapy) OR (Chemotherapy) OR (Adjuvant Therapy) OR (Radiotherapy) OR (External Radiotherapy) OR (Internal Radiotherapy) OR (Stereotactic Body Radiation Therapy) OR (Selective Internal Radiation Therapy) OR (SBRT) OR (Transarterial radioembolization) OR (CAR-T) OR (imaging) OR (Computed tomography) OR (CT) OR (Magnetic resonance imaging) OR (MRI) OR (Positron emission tomography) OR (PET) OR (ultrasound)

**#3:** #1 AND #2

Indexes=SCI-EXPANDED.

Timespan= From January 2000 to November 2021.

Note: SCI-EXPANDED, Science Citation Index Expanded.
