# Supplementary material for: Evolutions in the management of non-small cell lung cancer: A bibliometric study from the 100 most impactful articles in the field
Source: Front Oncol. 2022 Aug 17;12:939838. doi: 10.3389/fonc.2022.939838 (PMC9428518; doi:10.3389/fonc.2022.939838)
Supplement: Supplementary file 1 [file DataSheet_1.zip › Additional files/Supplement Table S1-S3/Supplementary Table S2.docx]

**Table S2 |** The top 100 most-cited publications in non-small cell lung cancer management.

| **Rank** | **Article** | **TC** |
| --- | --- | --- |
| 1 | EGFR mutations in lung cancer: Correlation with clinical response to gefitinib therapy | 7340 |
| 2 | Gefitinib or Carboplatin-Paclitaxel in Pulmonary Adenocarcinoma. | 5931 |
| 3 | Nivolumab versus Docetaxel in Advanced Nonsquamous Non-Small-Cell Lung Cancer | 5453 |
| 4 | Pembrolizumab versus Chemotherapy for PD-L1-Positive Non-Small-Cell Lung Cancer | 4777 |
| 5 | Mutational landscape determines sensitivity to PD-1 blockade in non-small cell lung cancer | 4526 |
| 6 | Paclitaxel-carboplatin alone or with bevacizumab for non-small-cell lung cancer | 4484 |
| 7 | Erlotinib in previously treated non-small-cell lung cancer | 4431 |
| 8 | Nivolumab versus Docetaxel in Advanced Squamous-Cell Non-Small-Cell Lung Cancer | 4391 |
| 9 | Early Palliative Care for Patients with Metastatic Non-Small-Cell Lung Cancer | 4074 |
| 10 | Comparison of four chemotherapy regimens for advanced non-small-cell lung cancer | 4052 |
| 11 | Gefitinib or Chemotherapy for Non-Small-Cell Lung Cancer with Mutated EGFR. | 3905 |
| 12 | Erlotinib versus standard chemotherapy as first-line treatment for European patients with advanced EGFR mutation-positive non-small-cell lung cancer (EURTAC): a multicentre, open-label, randomised phase 3 trial | 3730 |
| 13 | Pembrolizumab for the Treatment of Non-Small-Cell Lung Cancer | 3580 |
| 14 | Anaplastic Lymphoma Kinase Inhibition in Non-Small-Cell Lung Cancer | 3324 |
| 15 | Pembrolizumab versus docetaxel for previously treated, PD-L1-positive, advanced non-small-cell lung cancer (KEYNOTE-010): a randomised controlled trial | 3003 |
| 16 | Gefitinib versus cisplatin plus docetaxel in patients with non-small-cell lung cancer harbouring mutations of the epidermal growth factor receptor (WJTOG3405): an open label, randomised phase 3 trial | 2981 |
| 17 | Erlotinib versus chemotherapy as first-line treatment for patients with advanced EGFR mutation-positive non-small-cell lung cancer (OPTIMAL, CTONG-0802): a multicentre, open-label, randomised, phase 3 study | 2868 |
| 18 | Phase III study comparing cisplatin plus gemcitabine with cisplatin plus pemetrexed in chemotherapy-naive patients with advanced-stage non-small-cell lung cancer | 2449 |
| 19 | Atezolizumab versus docetaxel in patients with previously treated non-small-cell lung cancer (OAK): a phase 3, open-label, multicentre randomised controlled trial | 2402 |
| 20 | Crizotinib versus Chemotherapy in Advanced ALK-Positive Lung Cancer | 2400 |
| 21 | Pembrolizumab plus Chemotherapy in Metastatic Non-Small-Cell Lung Cancer | 2390 |
| 22 | Multi-institutional randomized phase II trial of gefitinib for previously treated patients with advanced non-small-cell lung cancer | 2376 |
| 23 | Efficacy of gefitinib, an inhibitor of the epidermal growth factor receptor tyrosine kinase, in symptomatic patients with non-small cell lung cancer - A randomized trial | 2109 |
| 24 | Phase III Study of Afatinib or Cisplatin Plus Pemetrexed in Patients With Metastatic Lung Adenocarcinoma With EGFR Mutations | 2068 |
| 25 | Non-small cell lung cancer: Epidemiology, risk factors, treatment, and survivorship | 2049 |
| 26 | First-Line Crizotinib versus Chemotherapy in ALK-Positive Lung Cancer | 1963 |
| 27 | Randomized phase III trial of pemetrexed versus docetaxel in patients with non-small-cell lung cancer previously treated with chemotherapy | 1895 |
| 28 | Osimertinib in Untreated EGFR-Mutated Advanced Non-Small-Cell Lung Cancer | 1759 |
| 29 | Durvalumab after Chemoradiotherapy in Stage III Non-Small-Cell Lung Cancer | 1755 |
| 30 | Prospective randomized trial of docetaxel versus best supportive care in patients with non-small-cell lung cancer previously treated with platinum-based chemotherapy | 1733 |
| 31 | Screening for Epidermal Growth Factor Receptor Mutations in Lung Cancer. | 1723 |
| 32 | Gefitinib plus best supportive care in previously treated patients with refractory advanced non-small-cell lung cancer: results from a randomised, placebo-controlled, multicentre study (Iressa Survival Evaluation in Lung Cancer) | 1720 |
| 33 | Stereotactic Body Radiation Therapy for Inoperable Early Stage Lung Cancer | 1715 |
| 34 | Cisplatin-based adjuvant chemotherapy in patients with completely resected non-small-cell lung cancer | 1669 |
| 35 | Osimertinib or Platinum-Pemetrexed in EGFR T790M-Positive Lung Cancer | 1594 |
| 36 | Randomized phase II trial comparing bevacizumab plus carboplatin and paclitaxel with carboplatin and paclitaxel alone in previously untreated locally advanced or metastatic non-small-cell lung cancer | 1526 |
| 37 | Erlotinib in lung cancer - Molecular and clinical predictors of outcome | 1511 |
| 38 | Atezolizumab for First-Line Treatment of Metastatic Nonsquamous NSCLC | 1451 |
| 39 | Nivolumab plus Ipilimumab in Lung Cancer with a High Tumor Mutational Burden | 1405 |
| 40 | DNA repair by ERCC1 in non-small-cell lung cancer and cisplatin-based adjuvant chemotherapy | 1389 |
| 41 | Atezolizumab versus docetaxel for patients with previously treated non-small-cell lung cancer (POPLAR): a multicentre, open-label, phase 2 randomised controlled trial | 1380 |
| 42 | Gefitinib in combination with gemcitabine and cisplatin in advanced non-small-cell lung cancer: A phase III trial-INTACT1 | 1379 |
| 43 | Lung Adjuvant Cisplatin Evaluation: A pooled analysis by the LACE collaborative group | 1368 |
| 44 | AZD9291 in EGFR Inhibitor-Resistant Non-Small-Cell Lung Cancer | 1364 |
| 45 | Gefitinib in combination with paclitaxel and carboplatin in advanced non-small-cell lung cancer: A phase III trial - INTACT 2 | 1364 |
| 46 | Vinorelbine plus cisplatin vs. observation in resected non-small-cell lung cancer | 1352 |
| 47 | First-Line Nivolumab in Stage IV or Recurrent Non-Small-Cell Lung Cancer | 1323 |
| 48 | Epidermal growth factor receptor gene and protein and gefitinib sensitivity in non-small-cell lung cancer | 1320 |
| 49 | Afatinib versus cisplatin plus gemcitabine for first-line treatment of Asian patients with advanced non-small-cell lung cancer harbouring EGFR mutations (LUX-Lung 6): an open-label, randomised phase 3 trial | 1309 |
| 50 | Pembrolizumab plus Chemotherapy for Squamous Non-Small-Cell Lung Cancer | 1296 |
| 51 | Mutations in the epidermal growth factor receptor and in KRAS are predictive and prognostic indicators in patients with non-small-cell lung cancer treated with chemotherapy alone and in combination with erlotinib | 1172 |
| 52 | TRIBUTE: A phase III trial of erlotinib hydrochloride (OSI-774) combined with carboplatin and paclitaxel chemotherapy in advanced non-small-cell lung cancer | 1164 |
| 53 | Crizotinib in ROS1-Rearranged Non-Small-Cell Lung Cancer | 1149 |
| 54 | Phase III Trial of Cisplatin Plus Gemcitabine With Either Placebo or Bevacizumab As First-Line Therapy for Nonsquamous Non-Small-Cell Lung Cancer: AVAiL | 1147 |
| 55 | Standard-dose versus high-dose conformal radiotherapy with concurrent and consolidation carboplatin plus paclitaxel with or without cetuximab for patients with stage IIIA or IIIB non-small-cell lung cancer (RTOG 0617): a randomised, two-by-two factorial phase 3 study | 1136 |
| 56 | The biology and management of non-small cell lung cancer | 1112 |
| 57 | Meta-Analysis of Concomitant Versus Sequential Radiochemotherapy in Locally Advanced Non-Small-Cell Lung Cancer | 1097 |
| 58 | Adjuvant vinorelbine plus cisplatin versus observation in patients with completely resected stage IB-IIIA non-small-cell lung cancer (Adjuvant Navelbine International Trialist Association [ANITA]): a randomised controlled trial | 1092 |
| 59 | Overall Survival with Durvalumab after Chemoradiotherapy in Stage III NSCLC | 1085 |
| 60 | Ceritinib in ALK-Rearranged Non-Small-Cell Lung Cancer | 1085 |
| 61 | Cetuximab plus chemotherapy in patients with advanced non-small-cell lung cancer (FLEX): an open-label randomised phase III trial | 1071 |
| 62 | Randomized phase III trial of docetaxel versus vinorelbine or ifosfamide in patients with advanced non-small-cell lung cancer previously treated with platinum-containing chemotherapy regimens | 1054 |
| 63 | Gefitinib versus docetaxel in previously treated non-small-cell lung cancer (INTEREST): a randomised phase III trial | 1050 |
| 64 | Excessive toxicity when treating central tumors in a phase II study of stereotactic body radiation therapy for medically inoperable early-stage lung cancer | 1024 |
|  |  |  |
| 65 | Afatinib versus cisplatin-based chemotherapy for EGFR mutation-positive lung adenocarcinoma (LUX-Lung 3 and LUX-Lung 6): analysis of overall survival data from two randomised, phase 3 trials | 1022 |
| 66 | Biomarker Analyses and Final Overall Survival Results From a Phase III, Randomized, Open-Label, First-Line Study of Gefitinib Versus Carboplatin/Paclitaxel in Clinically Selected Patients With Advanced Non-Small-Cell Lung Cancer in Asia (IPASS) | 1016 |
| 67 | Pembrolizumab versus chemotherapy for previously untreated, PD-L1-expressing, locally advanced or metastatic non-small-cell lung cancer (KEYNOTE-042): a randomised, open-label, controlled, phase 3 trial | 1010 |
| 68 | Alectinib versus Crizotinib in Untreated ALK-Positive Non-Small-Cell Lung Cancer | 998 |
| 69 | Activity and safety of nivolumab, an anti-PD-1 immune checkpoint inhibitor, for patients with advanced, refractory squamous non-small-cell lung cancer (CheckMate 063): a phase 2, single-arm trial | 981 |
| 70 | Erlotinib as maintenance treatment in advanced non-small-cell lung cancer: a multicentre, randomised, placebo-controlled phase 3 study | 960 |
| 71 | Radiotherapy plus chemotherapy with or without surgical resection for stage III non-small-cell lung cancer: a phase III randomised controlled trial | 940 |
| 72 | Activity and safety of crizotinib in patients with ALK-positive non-small-cell lung cancer: updated results from a phase 1 study | 920 |
| 73 | Carboplatin and pemetrexed with or without pembrolizumab for advanced, non-squamous non-small-cell lung cancer: a randomised, phase 2 cohort of the open-label KEYNOTE-021 study | 900 |
| 74 | Maintenance pemetrexed plus best supportive care versus placebo plus best supportive care for non-small-cell lung cancer: a randomised, double-blind, phase 3 study | 869 |
| 75 | Determinants of tumor response and survival with erlotinib in patients with non-small-cell lung cancer | 869 |
| 76 | Randomized phase III trial of paclitaxel plus carboplatin versus vinorelbine plus cisplatin in the treatment of patients with advanced non-small-cell lung cancer: A Southwest Oncology Group trial | 865 |
| 77 | Stereotactic ablative radiotherapy versus lobectomy for operable stage I non-small-cell lung cancer: a pooled analysis of two randomised trials | 836 |
| 78 | Mutations of the epidermal growth factor receptor gene predict prolonged survival after gefitinib treatment in patients with non-small-cell lung cancer with postoperative recurrence | 794 |
| 79 | Randomized, multinational, phase III study of docetaxel plus platinum combinations versus vinorelbine plus cisplatin for advanced non-small-cell lung cancer: The TAX 326 study group | 735 |
| 80 | Afatinib versus placebo for patients with advanced, metastatic non-small-cell lung cancer after failure of erlotinib, gefitinib, or both, and one or two lines of chemotherapy (LUX-Lung 1): a phase 2b/3 randomised trial | 727 |
|  |  |  |
| 81 | Sequential vs Concurrent Chemoradiation for Stage III Non-Small Cell Lung Cancer: Randomized Phase III Trial RTOG 9410 | 727 |
| 82 | Phase III study of erlotinib in combination with cisplatin and gemcitabine in advanced non-small-cell lung cancer: The Tarceva Lung Cancer Investigation Trial | 727 |
| 83 | Ipilimumab in Combination With Paclitaxel and Carboplatin As First-Line Treatment in Stage IIIB/IV Non-Small-Cell Lung Cancer: Results From a Randomized, Double-Blind, Multicenter Phase II Study | 719 |
| 84 | Ramucirumab plus docetaxel versus placebo plus docetaxel for second-line treatment of stage IV non-small-cell lung cancer after disease progression on platinum-based therapy (REVEL): a multicentre, double-blind, randomised phase 3 trial | 711 |
| 85 | Effect of crizotinib on overall survival in patients with advanced non-small-cell lung cancer harbouring ALK gene rearrangement: a retrospective analysis | 691 |
| 86 | Hypofractionated stereotactic radiotherapy (HypoFXSRT) for stage I non-small cell lung cancer: Updated results of 257 patients in a Japanese multi-institutional study | 688 |
| 87 | Neoadjuvant PD-1 Blockade in Resectable Lung Cancer | 685 |
| 88 | Efficacy and safety of two doses of pemetrexed supplemented with folic acid and vitamin B-12 in previously treated patients with non-small cell lung cancer | 685 |
| 89 | Nivolumab plus Ipilimumab in Advanced Non-Small-Cell Lung Cancer | 679 |
| 90 | Phase III randomized trial comparing three platinum-based doublets in advanced non-small-cell lung cancer | 676 |
| 91 | First-line gefitinib in patients with advanced non-small-cell lung cancer harboring somatic EGFR mutations | 670 |
| 92 | Predictive and prognostic impact of epidermal growth factor receptor mutation in non-small-cell lung cancer patients treated with gefitinib | 651 |
| 93 | Adjuvant Paclitaxel Plus Carboplatin Compared With Observation in Stage IB Non-Small-Cell Lung Cancer: CALGB 9633 With the Cancer and Leukemia Group B, Radiation Therapy Oncology Group, and North Central Cancer Treatment Group Study Groups | 628 |
| 94 | A phase I study of dexosome immunotherapy in patients with advanced non-small cell lung cancer | 628 |
| 95 | Outcome in a Prospective Phase II Trial of Medically Inoperable Stage I Non-Small-Cell Lung Cancer Patients Treated With Stereotactic Body Radiotherapy | 627 |
| 96 | Chemotherapy for elderly patients with advanced non-small-cell lung cancer: The Multicenter Italian Lung Cancer in the Elderly Study (MILES) phase III randomized trial | 625 |
| 97 | Non-small cell lung cancer: current treatment and future advances | 623 |
| 98 | Stereotactic hypofractionated high-dose irradiation for stage I nonsmall cell lung carcinoma - Clinical outcomes in 245 subjects in a Japanese multinstitutional study | 623 |
|  |  |  |
| 99 | Afatinib versus gefitinib as first-line treatment of patients with EGFR mutation-positive non-small-cell lung cancer (LUX-Lung 7): a phase 2B, open-label, randomised controlled trial | 618 |
| 100 | Molecular Determinants of Response to Anti-Programmed Cell Death (PD)-1 and Anti-Programmed Death-Ligand 1 (PD-L1) Blockade in Patients With Non-Small-Cell Lung Cancer Profiled With Targeted Next-Generation Sequencing | 615 |

Note: TC, total citation.
