# Supplementary material for: Evolutions in the management of non-small cell lung cancer: A bibliometric study from the 100 most impactful articles in the field
Source: Front Oncol. 2022 Aug 17;12:939838. doi: 10.3389/fonc.2022.939838 (PMC9428518; doi:10.3389/fonc.2022.939838)
Supplement: Supplementary file 1 [file DataSheet_1.zip › Additional files/Supplement Table S1-S3/Supplementary Table S3.docx]

**Table S3 |** Countries/regions that published the top-cited 100 articles.

| **Rank** | **Country** | **Publication** | **TC** | **TC/Publication** |
| --- | --- | --- | --- | --- |
| 1 | USA | 76 | 133584 | 1757.68 |
| 2 | Germany | 34 | 56351 | 1657.38 |
| 3 | France | 33 | 55215 | 1673.18 |
| 4 | South Korea | 32 | 47064 | 1470.75 |
| 5 | Spain | 29 | 57259 | 1974.45 |
| 6 | Italy | 27 | 47501 | 1759.30 |
| 7 | Japan | 27 | 56451 | 2090.78 |
| 8 | England | 25 | 42793 | 1711.72 |
| 9 | Canada | 24 | 43607 | 1816.96 |
| 10 | Australia | 22 | 37781 | 1717.32 |
| 11 | China | 21 | 33728 | 1606.10 |
| 12 | Poland | 19 | 25773 | 1356.47 |
| 13 | Netherlands | 13 | 26454 | 2034.92 |
| 14 | Russia | 13 | 21690 | 1668.46 |
| 15 | Brazil | 11 | 20677 | 1879.73 |
| 16 | Belgium | 9 | 14285 | 1587.22 |
| 17 | Thailand | 9 | 20266 | 2251.78 |
| 18 | Hungary | 8 | 13174 | 1646.75 |
| 19 | Romania | 8 | 12098 | 1512.25 |
| 20 | Switzerland | 8 | 9118 | 1139.75 |
| 21 | Turkey | 8 | 11805 | 1475.63 |
| 22 | Singapore | 7 | 11492 | 1641.71 |
| 23 | Chile | 6 | 14321 | 2386.83 |
| 24 | India | 5 | 6468 | 1293.60 |
| 25 | Sweden | 5 | 5160 | 1032.00 |
| 26 | Austria | 4 | 5719 | 1429.75 |
| 27 | Czech Republic | 4 | 7588 | 1897.00 |
| 28 | Greece | 4 | 3887 | 971.75 |
| 29 | Ireland | 4 | 10316 | 2579.00 |
| 30 | Mexico | 4 | 8139 | 2034.75 |
| 31 | Argentina | 3 | 5821 | 1940.33 |
| 32 | Denmark | 3 | 4126 | 1375.33 |
| 33 | Ukraine | 3 | 2800 | 933.33 |
| 34 | Finland | 2 | 2468 | 1234.00 |
| 35 | Israel | 2 | 7167 | 3583.50 |
| 36 | South Africa | 2 | 4131 | 2065.50 |
| 37 | Bulgaria | 1 | 869 | 869.00 |
| 38 | Croatia | 1 | 869 | 869.00 |
| 39 | Indonesia | 1 | 5931 | 5931.00 |
| 40 | Latvia | 1 | 1010 | 1010.00 |
| 41 | Lithuania | 1 | 960 | 960.00 |
| 42 | Malaysia | 1 | 1759 | 1759.00 |
| 43 | Norway | 1 | 627 | 627.00 |
| 44 | Pakistan | 1 | 1895 | 1895.00 |

Note: TC, total citation.
