# Supplementary figures and images for: Evolutions in the management of non-small cell lung cancer: A bibliometric study from the 100 most impactful articles in the field
Source: Front Oncol. 2022 Aug 17;12:939838. doi: 10.3389/fonc.2022.939838 (PMC9428518; doi:10.3389/fonc.2022.939838)

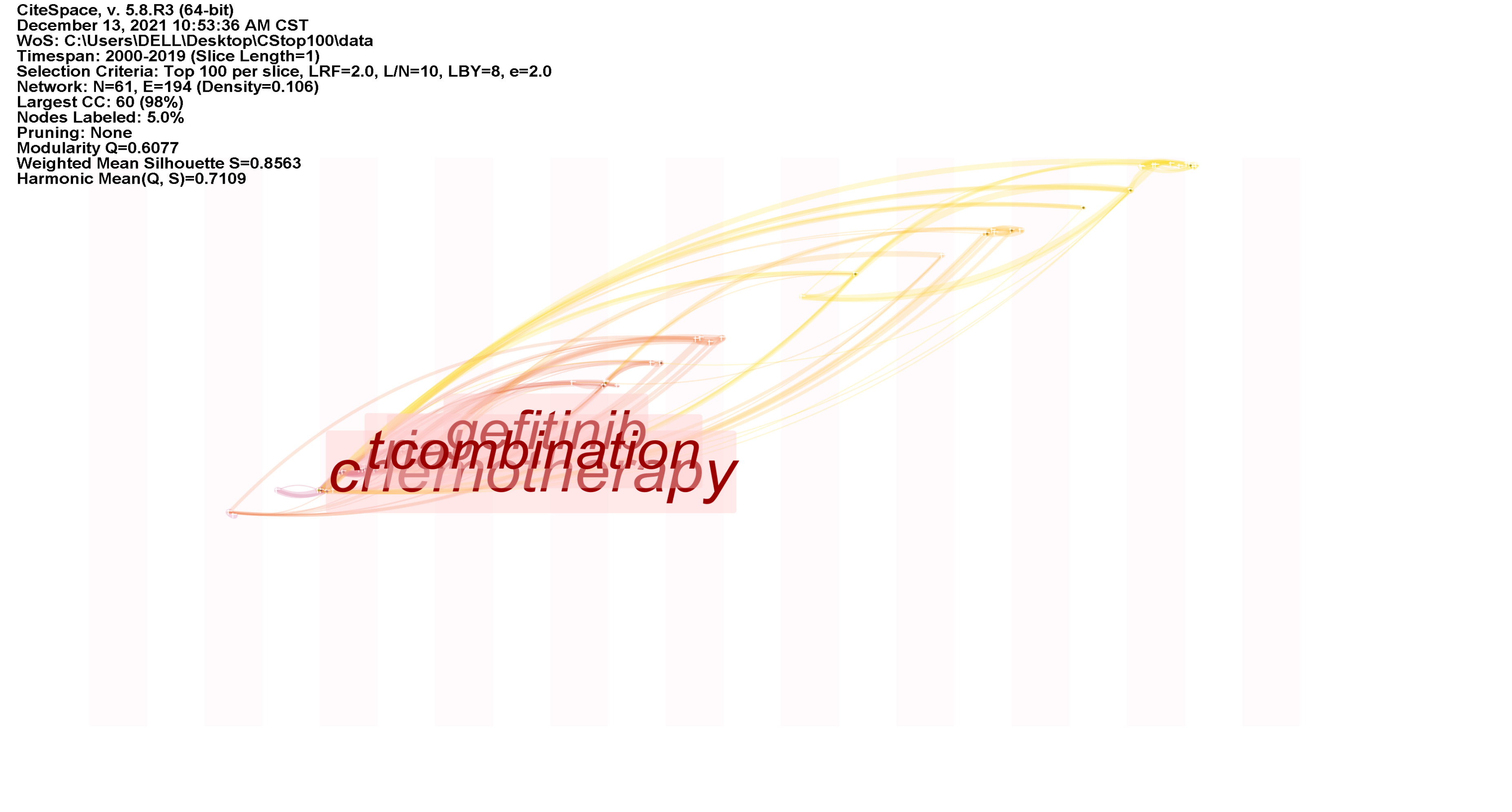

Supplement: Supplementary file 1 [file DataSheet_1.zip › Additional files/Figure production/CiteSpace figure production/project/COA_v61e194.png]
